# An Extensive Circuitry for Cell Wall Regulation in *Candida albicans*

**DOI:** 10.1371/journal.ppat.1000752

**Published:** 2010-02-05

**Authors:** Jill R. Blankenship, Saranna Fanning, Jessica J. Hamaker, Aaron P. Mitchell

**Affiliations:** 1 Department of Biological Sciences, Carnegie Mellon University, Pittsburgh, Pennsylvania, United States of America; 2 Department of Microbiology and Institute of Cancer Research, Columbia University, New York, New York, United States of America; 3 Department of Microbiology, University College Cork, Cork, Ireland; University of Wisconsin, -Madison, United States of America

## Abstract

Protein kinases play key roles in signaling and response to changes in the external environment. The ability of *Candida albicans* to quickly sense and respond to changes in its environment is key to its survival in the human host. Our guiding hypothesis was that creating and screening a set of protein kinase mutant strains would reveal signaling pathways that mediate stress response in *C. albicans*. A library of protein kinase mutant strains was created and screened for sensitivity to a variety of stresses. For the majority of stresses tested, stress response was largely conserved between *C. albicans*, *Saccharomyces cerevisiae*, and *Schizosaccharomyces pombe*. However, we identified eight protein kinases whose roles in cell wall regulation (CWR) were not expected from functions of their orthologs in the model fungi *Saccharomyces cerevisiae* and *Schizosaccharomyces pombe*. Analysis of the conserved roles of these protein kinases indicates that establishment of cell polarity is critical for CWR. In addition, we found that septins, crucial to budding, are both important for surviving and are mislocalized by cell wall stress. Our study shows an expanded role for protein kinase signaling in *C. albicans* cell wall integrity. Our studies suggest that in some cases, this expansion represents a greater importance for certain pathways in cell wall biogenesis. In other cases, it appears that signaling pathways have been rewired for a cell wall integrity response.

## Author Summary

*Candida albicans* is the major fungal commensal and pathogen of humans. Like most microorganisms, *C. albicans* is surrounded and protected by a cell wall. This cell wall has two purposes: to act as a rigid “exoskeleton” to prevent cells from bursting, and to provide a surface where a cell can interact with the outside environment while protecting the cell itself from this environment. Maintenance of this structure has been well studied in the model fungus, *Saccharomyces cerevisiae*, but previous evidence suggested that *C. albicans* might have additional inputs to this process. By creating and testing a library of mutant strains for sensitivity to cell wall stress, we were able to identify a number of conserved genes with roles in this process not shared by their *S. cerevisiae* counterparts. Although some of these genes had previously been linked to cell wall integrity, it appears that they have increased impact on this process in *C. albicans*. For other genes, their role in cell wall integrity may represent a novel connection. Our findings provide insight into novel aspects of cell wall integrity in this pathogen and lay a foundation for its more detailed analysis.

## Introduction

The cell surface has two pivotal roles in the survival of microorganisms: protection and interaction. The protective role preserves the integrity of the cell in the face of environmental assaults [1],[2]. The interactive role includes both sensing of external signals and binding to other cells or external surfaces [3],[4]. These two functions necessitate a delicate balance for organisms that can adapt to diverse niches: protection is accomplished by a rigid and impermeable surface; interaction is accomplished by a dynamic one.

The fungus *Candida albicans* has considerable adaptive ability that is manifested through its impact on humans. It is a commensal microbe that is acquired soon after birth, and occupies both the GI and GU tracts. These colonization sites represent distinct environments in terms of cohabitant microbiota, pH, and nutrients. The adaptive ability of *C. albicans* is most apparent when circumstances compromise host barriers to infection. Immunological deficiency or presence of an implanted device provides the opportunity for *C. albicans* to invade and grow in almost any tissue in the body. Hence *C. albicans* is the major fungal commensal and the major fungal pathogen of humans.

*C. albicans* is protected by a carbohydrate-based cell wall, whose major constituent is β-glucan, a glucose polymer that imparts shape [5]. Chitin lends the wall rigidity, while mannoproteins that coat the surface serve to interact with the external environment [6]. The cell wall is connected to every known *C. albicans* biological process, including growth, morphogenesis, mating, and pathogenicity [7]. It is also an inviting therapeutic target, being a source of fungal-specific antigens and essential functions. Thus the mechanisms and regulatory pathways that govern *C. albicans* cell wall dynamics are a critical area of understanding.

Much of what we know about fungal cell wall integrity and cell wall biogenesis (herein collectively called cell wall regulation, CWR) comes from studies of the model yeast *Saccharomyces cerevisiae*, where the protein kinase C-MAPK (PKC-MAPK) pathway, largely responsible for the transcriptional response to cell wall disturbance, is a major cell wall-responsive regulatory system (reviewed in [8]). The PKC-MAPK pathway is also vital for cell wall integrity the pathogenic fungus *Cryptococcus neoformans* [9]. PKC itself has additional targets that impact cell wall structure independently of the MAPK pathway [10]–[13]. PKC-MAPK signaling is activated by cell wall disruption, or by diverse signals that include oxidative stress, hypo-osmotic shock, and mating projection formation. The spectrum of PKC-MAPK signaling inputs reflects the central role of the cell wall in *S. cerevisiae* biological processes.

The PKC-MAPK pathway is conserved in *C. albicans*, where it also has a significant role in CWR [14],[15]. However, several observations suggest that *C. albicans* cell wall dynamics may be under control of a broader signaling network [16],[17]. We have considered the set of *C. albicans* protein kinases (PKs) to be representatives of the spectrum of signaling pathways, and created a panel of mutant strains defective in PK genes and some PK-related genes. The panel has been used to connect PK function to CWR as well as such features as morphogenesis, biofilm formation, and stress sensitivity. Our findings reveal that the *C. albicans* cell wall is highly connected to a much broader range of signaling pathways than has been found for *S. cerevisiae*. The *C. albicans* cell wall signaling network may reflect a balance of diverse inputs that are poised to promote modification and support adaptation.

## Results

### Construction of a protein kinase (PK) mutant library

We set out to define functional networks that connect signal transduction pathways to signature *C. albicans* biological features. We created homozygous insertion or deletion mutations in 67 PK genes and 13 PK-related genes. More detail about these mutants, including insertion sites, can be found in Table S1. Most genes were represented by multiple independent mutant isolates. We were unable to recover homozygous mutations in 41 PK genes.

PK function was surveyed through a screen of the mutants for altered biological properties that included stress or drug sensitivity, filamentation, and biofilm formation (Table S1). Prior studies of *Saccharomyces cerevisiae* and *Schizosaccharomyces pombe* [18] provided clear hypotheses for the function of many PKs, and we generally found good correspondence with these predictions (Tables 1 and 2). However, there was a strikingly expanded role for *C. albicans* PKs in CWR: 24 of the 80 mutants were hypersensitive to the cell wall inhibitor caspofungin, compared to 10 of the mutants that were predicted by model organism studies (Table 1). In addition, 2 of the 10 *C. albicans* mutants predicted to be sensitive to cell wall stress based on the phenotypes of orthologous *S. cerevisiae* mutants were not sensitive in our assays.

**Table 1:** Comparison of *C. albicans*, *S. cerevisiae*, and *S. pombe* mutant PK and PK-related cell wall stress sensitivity phenotypes.

*C. a.* Gene name	*Cell wall stress phenotype* 1		
	*C. a.*	*S. c.* 2	*S. p.* 3
*BCK1*	−	−	+
*CBK1*	−	−	+
*CKB1*	−	+	+
*CKB2*	−	+	+
*CLA4*	−	−	+
*GIN4*	−	−	+
*HSL1*	−	+	+
*HST7*	−	+	+
*IRE1*	−	−	+
*KIN3*	−	−	+
*KIS1*	−	+	+
*MKC1*	−	−	−
*MKK2*	−	−	+
*MSS2*	−	−	N/A
*PKC1*	−	−	+
*PSK1*	−	−	+
*PRK1*	−	+	+
*RIO2*	−	+	+
*SIP3*	−	+	+
*SWE1*	−	+	−
*TPK1*	−	+	+
*VPS34*	−	−	+
*YCK2*	−	+	+
*YCK3*	−	+	+
*CKA2*	+	−	?
*CPP1*	+	−	?
*PKH1*	+	+	−
*RIM15*	+	+	−
*SAT4*	+	+	−
*KIN2*	+	+	−
*YAK1*	+	+	−
*PBS2*	+	+	−

1 Genes listed are required for resistance to cell wall stress in at least one of the fungi represented: *C. a. (C. albicans,) S. c. (S. cerevisiae), or S. p. (S. pombe)*. An entry of – means that a null mutation of this gene causes hypersensitivity to cell wall stress. An entry of + means that a null mutation of this gene does not alter sensitivity to cell wall stress. An entry of ? means the phenotype of the null mutation is unknown. Information for this table was gathered experimentally or from the Saccharomyces Genome Database (www.yeastgenome.org/), Biobase (portal.biobase-international.com/cgi-bin/portal/login.cgi), reference [18], and literature searches.

2 *S. cerevisiae* ortholog names do not always correspond to the *C. albicans* gene name. *HST7* is *ScSTE7*, *MKC1* is *ScSLT2*, and *CPP1* is *ScMSG5*. *PSK1* from *C. albicans* has two orthologs in *S. cerevisiae*, *PSK1* and *PSK2*. The cell wall stress hypersensitivity is only observed in the double mutant. The observation that the *Sckin3* and *Scmss4* mutant strains are hypersensitive to cell wall stress is first reported in this work.

3 Many of the gene names are different between *C. albicans* and *S. pombe*. *MKC1* is *SpSPM1*, *SWE1* is *SpWEE1*, *PKH1* is *SpKSG1*, *RIM15* is *SpCEK1*, *SAT4* is *SpHAL4*, *KIN2* is *SpKIN1*, *YAK1* is *SpPOM1*, *PBS2* is *SpWIS1*, *CKA2* is *SpCKA1*, and *CPP1* is *SpPMP1*. Mutations in *SpCKA1* and *SpPMP1* have not been tested for sensitivity to cell wall stress and are marked ? for unknown. There is no obvious *S. pombe* ortholog to *MSS2* and this cell is labeled N/A. The majority of *S. pombe* sensitivity assays were performed in SDS, which may impact more than just the cell wall.

**Table 2:** A comparison of *C. albicans*, *S. cerevisiae*, and *S. pombe* PK and PK-related mutant phenotypes.

*C. a.* gene name	*C. a.*	*S. c.* 1	*S. p.* 2
	**Oxidative stress phenotype** 3		
*CHK1*	−	+	+
*CKB1*	−	+	+
*GIN4*	−	+	+
*HOG1*	−	−	−
*KIN4*	−	+	+
*KIS1*	−	+	+
*PBS2*	−	−	−
*PRK1*	−	+	+
*SLN1*	−	+	+
*SSN3*	−	−	+
*SSN8*	−	−	+
*STE11*	−	+	+
*VPS34*	−	+	+
*MEC1*	+	−	?
*RCK2*	+	−	+
*PKT2*	+	−	?
	**Osmotic stress phenotype** 3		
*GIN4*	−	+	+
*HOG1*	−	−	−
*KIS1*	−	+	+
*PBS2*	−	−	−
*SOK1*	−	−	+
*VPS34*	−	−	−
*CKA2*	+	−	?
*CKB1*	+	−	?
*CKB2*	+	−	?
*HNT1*	+	−	?
*IES1*	+	−	N/A
*KSP1*	+	−	?
*MKC1*	+	−	?
*SWE1*	+	−	+
*SAT4*	+	+	−
*KIN2*	+	?	−
*BCK1*	+	+	−
*TPK2*	+	?	−
*CPP1*	+	?	−
*VPS15*	+	?	−
	**Filamentation/morphology phenotype** 3		
*CBK1*	−	+	−
*GIN4*	−	+	−
*IRE1*	−	+	+
*MSS2*	−	+	N/A
*RIO2*	−	+	+
*SOK1*	−	+	+
*TPK2*	−	−	+
*CLA4*	+	−	−
*KIS1*	+	−	?
*HST7*	+	−	?
*STE11*	+	−	?
*CST20*	+	−	−
*SWE1*	+	−	?
*CDC15*	+	?	−
*CKA2*	+	?	−
*KIN3*	+	?	−
*MEK1*	+	?	−
*PKC1*	+	?	−
*MEC1*	+	?	−

1 *S. cerevisiae* ortholog names do not always correspond to the *C. albicans* gene name. *MKC1* is *ScSLT2*, *CPP1* is *ScMSG5*, *KIS1* is *ScGAL83*, *HST7* is *Sc STE7*, and *CST20* is *ScSTE20*. The observation that *Scssn3* and *Scssn8* mutants are hypersensitive to oxidative stress is first reported in this work. For the morphology defect portion of the table, observed morphological defects of *S. cerevisiae* fall into one of three categories: invasive growth defect, pseudohyphal growth defect, or both. The *S. cerevisiae* orthologs of *TPK1*, *CLA4*, *KIS1*, *HST7*, and *CST20* are important for invasive growth. The *S. cerevisiae* orthologs of *TPK2*, *HST7*, *STE11*, *CST20*, and *SWE1* are important for pseudohyphal growth.

2 Many of the gene names are different between *C. albicans* and *S. pombe*. *HOG1* is *SpSTY1*, *PBS2* is *SpWIS1*, *MEC1* is *SpRAD3*, *RCK2* is *SpSRK1*, *PKT2* is most orthologous to *SpSPCC70.05c*, *CKA2* is *SpCKA1*, *HNT1* is *SPCC1442.14c*, *MKC1* is *SpSPM1*, *SWE1* is *SpWEE1*, *SAT4* is *SpHAL4*, *KIN2* is *SpKIN1*, *BCK1* is *SpMKH1*, *TPK2* is *PKA1*, *CPP1* is *SpPMP1*, *VPS15* is *SpPPK19*, *CBK1* is *SpORB6*, *GIN4* is *SpCDR2*, *CLA4* is *SpSHK2*, *KIS1* is *SpSPCC1919.03c*, *HST7* is *SpBYR1*, *STE11* is *SpBYR2*, *CDC15* is *SpCDC7*, *CKB1* is *SpCKB2*, *KIN3* is *SpFIN1*, *CST20* is *SpPAK1*, and *PKC1* is *SpPCK2*. N/A refers to *C. albicans* genes that do not have known orthologs in *S. pombe*. Under the osmotic stress category, sensitivities of the *Spkin2*, *Sptpk2* and *Spvps15* mutants were noted using KCl instead of NaCl.

3 Genes listed are required for resistance to the indicated stress in at least one of the fungi represented: *C. a. (C. albicans,) S. c. (S. cerevisiae),* or *S. p. (S. pombe)*. Oxidative stress generally refers to stress induced by H_2_O_2_, osmotic stress generally refers to stress induced by NaCl. Genes listed under filamentation/morphology phenotype are required for appropriate morphology or are required for changes in morphology. An entry of – means that a null mutation of this gene causes hypersensitivity to cell wall stress or defective morphology. An entry of + means that a null mutation of this gene does not alter sensitivity to cell wall stress or have normal morphology. An entry of ? means the phenotype of the null mutation is unknown. Information for this table was gathered experimentally or from the Saccharomyces Genome Database (www.yeastgenome.org/), Biobase (portal.biobase-international.com/cgi-bin/portal/login.cgi), reference [18], and literature searches.

**Figure 1:**
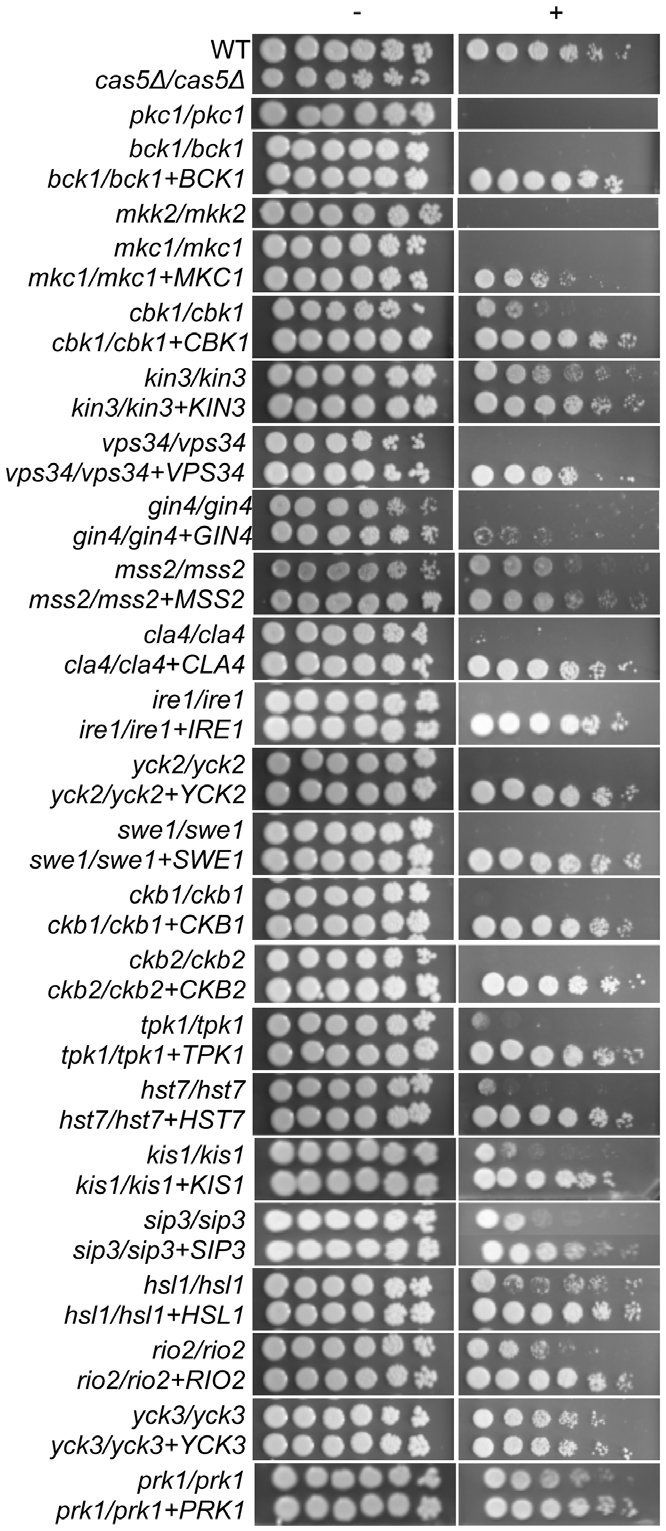
PKs play conserved and novel roles in CWR. A wild type marker-matched strain (DAY286), a hypersensitive *cas5Δ/cas5Δ* control, and the indicated prototrophic PK mutant strains and their complements were serially diluted onto YPD (−) or YPD+ 125 ng/ml caspofungin (+) and grown for 2 days at 30°C. Data for *PSK1* were published in Rauceo, et al [17]. In most cases, complementation fully restored growth on the caspofungin plates, but it should be noted that complementation of the *gin4−/−* strain with one copy of *GIN4* was not sufficient to restore growth on caspofungin.

PKs with conserved roles in CWR included members of the PKC MAPK pathway, as well as Cla4, Cbk1, Psk1, Gin4, Ire1, and Vps34 (Fig. 1, Table 1). However, the PKs Hsl1, Kin3, Swe1, Tpk1, Yck3, Prk1, Yck2, Rio2, and Hst7, along with the PK-related proteins Ckb1, Ckb2, Sip3, Mss2, and Kis1, were also required for normal sensitivity to caspofungin (Fig. 1). These genes had not previously been uncovered in large-scale *S. cerevisiae* screens for CWR, although a role for Hst7 in *C. albicans* CWR was previously identified [19]. Our own caspofungin-sensitivity tests of *S. cerevisiae* mutants lacking the respective orthologs revealed new roles in CWR for *S. cerevisiae* Kin3 and Mss2 (data not shown), but not for the other orthologs. Complementation of each new *C. albicans* caspofungin-hypersensitive mutant restored normal sensitivity to caspofungin (Fig. 1). Thus the defined mutation causes the CWR defect. Therefore, among the PKs and related proteins surveyed, we found 12 with conserved roles in CWR and 12 with apparent *C. albicans*-specific roles in CWR.

### The cell wall PK network is unique

We also surveyed PK function in a panel of tests for traits related to survival and virulence. The mutants were tested for sensitivity to oxidative, osmotic, and pH stress, as well as for the ability to form filamentous cells and biofilms. The results of these screens are shown in Table 2 and Fig. S1. While these additional screens demonstrated both conserved and novel roles for PKs in these responses, only the oxidative stress assay uncovered a significant expansion of roles among conserved PKs. Five PK genes (*PRK1*, *KIN4*, *VPS34*, *STE11*, and *GIN4*) and two PK-related genes (*CKB1* and *KIS1*) had previously undescribed roles in the oxidative stress response (Table 2 and Fig. S1). Analysis of *S. cerevisiae* mutants defective in their orthologs confirmed that their requirement for oxidative stress survival is unique to *C. albicans* (data not shown). This expanded role may reflect frequent encounters of *C. albicans* with host phagocytic cells that use oxidative attack during commensal growth. Rather than developing entirely new signaling pathways to combat this frequent stress, it appears that conserved pathways were enlisted to improve this fungus's chances for survival *in vivo*. The identification of almost twice the number of PKs with novel roles in CWR suggests that cell wall damage is also an important *in vivo* stress.

### PK mutants evoke a cell wall damage response in the absence of stress

**Figure 2:**
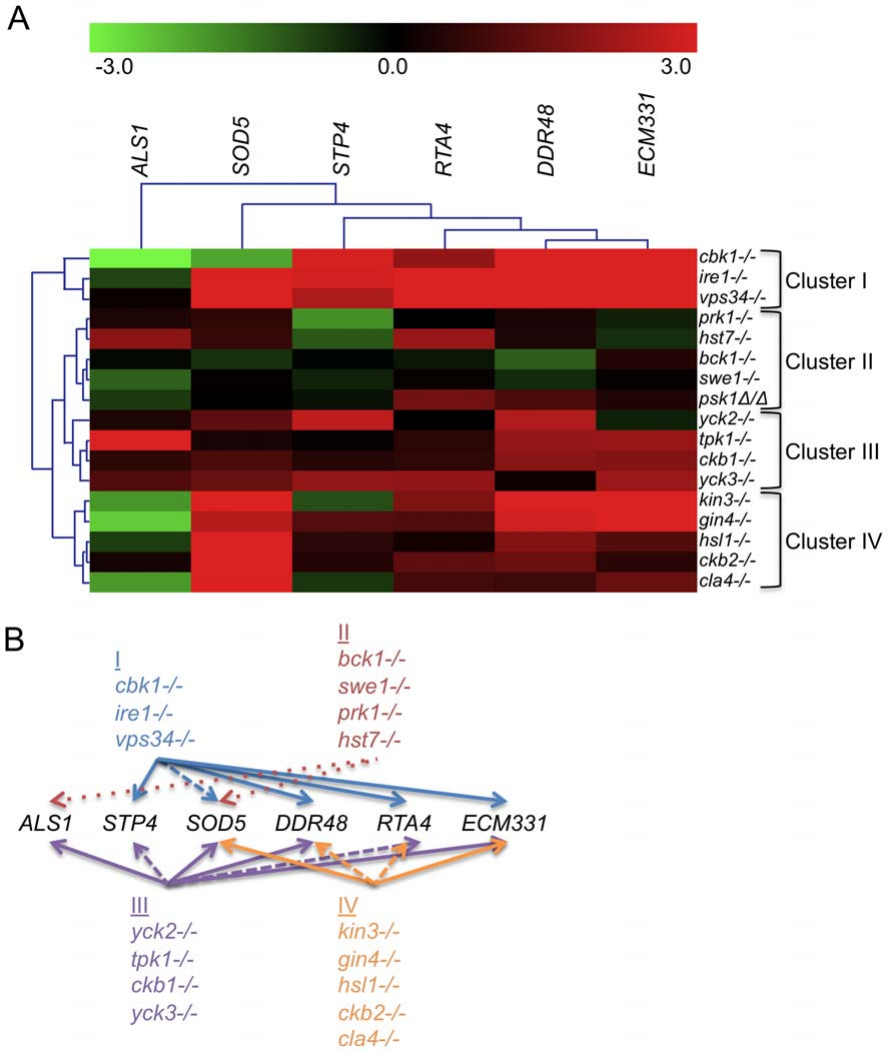
PK and PK-related mutant strains show a damage response in the absence of cell wall stress. (A) The expression of six genes upregulated by caspofungin treatment, *DDR48*, *SOD5*, *STP4*, *ALS1*, *RTA4*, and *ECM331*, was analyzed in PK and PK-related mutant strains in the absence of cell well stress. The expression of *TDH3*, a gene involved in glycolysis, was used to normalize expression between strains and expression values were further normalized to wild type (DAY185) expression for comparison between experiments. Resultant values were log base 2 transformed (wild type expression for all six genes is therefore at 0). (B) A graphical representation of the expression data. Arrows point to targets upregulated in all (solid arrows), most (dashed lines), or half (dotted lines) of the mutants indicated in the clusters.

We sought to determine whether the PK and PK-related genes with novel roles in CWR (Table 1) are required specifically for the response to cell wall damage, or have a role in cell wall biogenesis. Indeed, some of the conserved CWR PKs that appeared in our screens such as Cbk1, are important for cell wall biogenesis [20],[21]. We hypothesized that such mutants would exhibit a cell wall damage response even in the absence of exogenous cell wall stress. We therefore monitored the transcription of six cell wall damage response genes in the absence of externally induced cell wall stress in PK mutant and complement strains (Fig. 2 and S2). Two of these cell wall damage response genes, *DDR48* and *SOD5*, exhibit the highest upregulation following cell wall stress in previous microarray studies [17] and are also upregulated in hyphal growth and in the presence of other stresses. The regulation of *RTA4* is tied to both cell wall stress and to external pH [16],[22]. *ALS1*, an adhesin, is upregulated during biofilm formation and cell wall stress while *STP4* and *ECM331* appear to be more specific for caspofungin response [16],[23],[24].

Our first hypothesis, that strains mutant for genes involved in cell wall biogenesis will exhibit a damage profile in the absence of stress, is supported by the transcription profile from the *cbk1−/−* mutant (Fig. 2 and S2). We therefore tested the rest of the caspofungin-sensitive PK mutants for a damage response in the absence of cell wall stress by quantitative rtPCR (QrtPCR). Many of the PK mutant strains tested did display distinctive damage profiles compared to wild type and their respective complemented strains (data not shown). Hierarchical clustering of the QrtPCR data produced groupings of strains that may give us insight into shared functions between the PK and PK-related genes within the clusters. Cbk1 is important for cell wall biogenesis [20],[21] and the *cbk1−/−* mutant is in Cluster I. This cluster also contains the *ire1−/−* mutant, which had an even more pronounced damage profile than did the *cbk1−/−* mutant, and the *vps34−/−* mutant (Fig. 2). The magnitude of the damage response in Cluster I was significantly higher than other clusters (Fig. S2). We hypothesize that Cluster I represents PKs with intrinsic roles in cell wall biogenesis. Cluster II, representing mutant strains with very little difference in expression compared to the wild type strain, included the *bck1−/−* mutant. Bck1 is a member of the Pkc1 MAP kinase cascade, which is involved in sensing cell wall stress and we hypothesize that other members of this cluster may also play roles in cell wall stress response. These PKs may govern induction of caspofungin-inducible genes that we have yet to test, or may have posttranslational roles in CWR. This QrtPCR analysis included only a small subset of genes downstream of cell wall stress, and more detailed analysis will likely be required to tease out their potential roles in cell wall stress response. Thus, our gene expression analysis not only suggests that many of the PK and PK-related genes identified in our study have some role in cell wall biogenesis, but also has separated these genes into clusters that may reflect function.

### Relationship between septins and CWR

**Figure 3:**
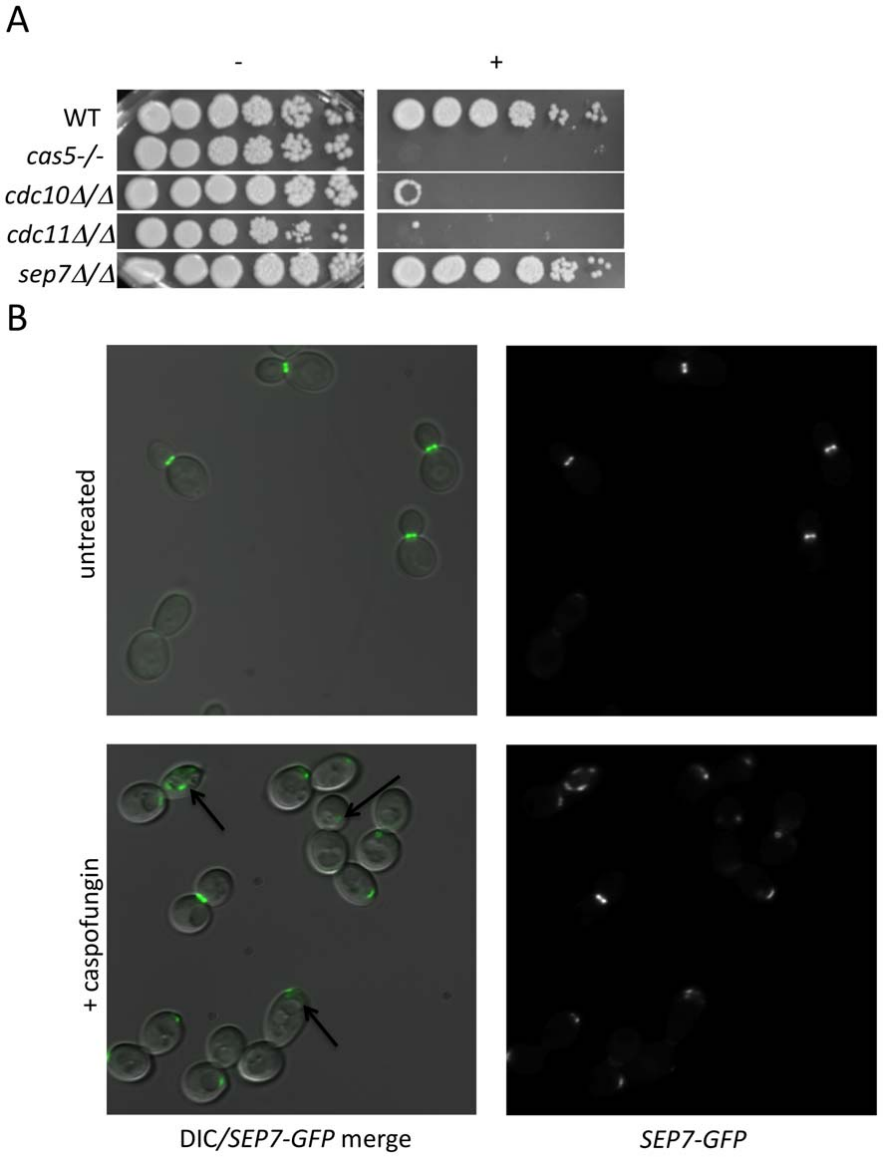
Septins play an integral role in CWR. (A) A wild type marker-matched strain (DAY185), a *cas5Δ/cas5Δ* negative control, a *cdc10Δ/cdc10Δ* (YAW7), a *cdc11Δcdc11/Δ* (YAW11), and a *sep7Δ/sep7Δ* (YAW41) mutant strain were serially diluted onto YPD (−) or YPD+ 125 ng/ml caspofungin (+) and grown for 3 days at 30°C. (B) *SEP7-GFP* cells (JRB217) were grown to log phase and either treated with 125 ng/ml caspofungin (+ caspofungin) for 30 minutes or left untreated. The cells were then visualized on glass slides. Arrows point to aberrant septin localization.

Cluster IV, identified in the previous analysis, contains several PKs with known connections to polar septin localization, including Cla4, Gin4, and Hsl1 [25],[26]. We hypothesized that septin function itself may be related to CWR. Two further observations support this hypothesis. First, we found that mutants defective in the septin genes *cdc10* and *cdc11* demonstrated increased sensitivity to caspofungin (Fig. 3A). These two septins are important for septin ring formation at high temperatures and chitin deposition at all temperatures [27]. In contrast, a *sep7* deletion mutant, which causes no apparent defect in yeast-form septin ring formation or chitin deposition, does not have heightened sensitivity to caspofungin. Severity of sensitivity thus correlated with the severity of the septation defects (Fig. 3A), an indication that septin function is vital for *C. albicans* survival during cell wall stress. Second, we asked whether cell wall disruption might alter septin localization. A Sep7-GFP fusion protein is concentrated at mother-bud necks in untreated budded cells, with two rings visible in separating mother-daughter cells (Fig. 3B), as previously reported [28]. Caspofungin treatment affected this localization dramatically: mislocalization was observed on one side of the mother-bud neck or in other regions of 46.7% of cells, while there were partial septa in 3.3% of budded cells (N = 92) (Fig. 3B). Caspofungin treatment of *CDC10-GFP*, *CDC12-GFP*, and *CDC3-GFP* strains caused similar abnormalities (data not shown). Therefore, septin localization depends upon normal CWR.

**Figure 4:**
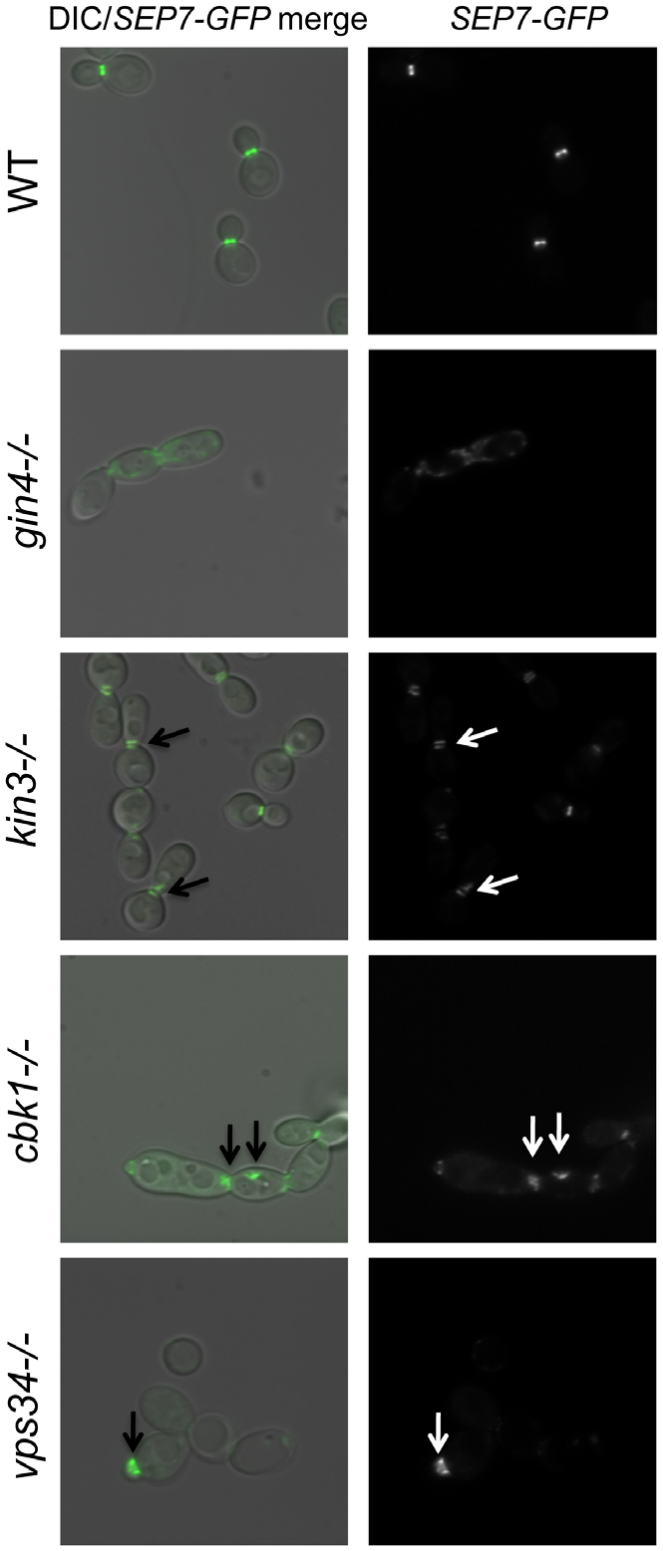
Septins are mislocalized in some PK mutant strains. *SEP7-GFP* tagged wild type (JRB217), *gin4−/−* (JRB221), *kin3−/−* (JRB193), *cbk1−/−* (JRB224), and *vps34−/−* (JRB216) strains were grown to log phase and imaged at 100× on glass slides. An overlay of DIC and GFP is on the left and GFP alone is on the right. Arrows point to aberrant septin localization.

We were led to the discovery of a role for septins in CWR by the sensitive phenotype of PK mutants with known roles in septin localization. We hypothesized that these PK mutants might display aberrant septin localization even in the absence of stress. Mutations causing a complete delocalization of septins at the mother-bud neck would probably be lethal to the cell and thus would not have been recovered in our library. To determine whether any of the caspofungin-sensitive PK and PK-related mutant strains displayed aberrant septin localization, a GFP tag was inserted into the 3′ end of the *SEP7* gene at the endogenous *SEP7* locus. We were able to insert the *SEP7-GFP* tagged allele into all but two of the caspofungin-sensitive PK and PK-related mutant strains (*hst7−/−* and *psk1−/−*). Cluster IV mutant strains, particularly *gin4−/−* and *cla4−/−* are the clearest candidates for delocalization based on phenotypes of the corresponding *S. cerevisiae* mutants [25],[29]. Indeed, septin localization was disrupted in the *gin4−/−* mutant strain (Fig. 4 and S3). Two cell phenotypes were observed: cells with normal buds had normal septin localization, but cells growing as pseudohyphae were either lacking septin rings or had septin rings that did not completely span the neck. Neither the *cla4−/−* nor the *hsl1−/−* had delocalized septins in the absence of stress (data not shown). This is consistent with the known roles of Cla4 in localization of septins in hyphal cells [30] and Hsl1 in signaling downstream of septin localization. However, one member of Cluster IV with no previous links to septin organization, Kin3, did demonstrate a septin organization defect, suggesting that this PK is linked to septin localization as predicted by its inclusion in this cluster. Septins in this strain appear normal in buds until cytokinesis, where separation of septin rings is aberrant. In addition to these Cluster IV mutants, we also observed partial septin mislocalization in the *vps34−/−* and *cbk1−/−* mutant strains (Fig. 4 and S3). Whether septin disorganization contributes to the caspofungin sensitivity of these strains or it is a symptom of an intrinsic cell wall defect has not yet been determined. However, the disorganization of septins in these strains lends further support to the hypothesis that septin localization or regulation is linked to cell wall integrity.

## Discussion

**Figure 5:**
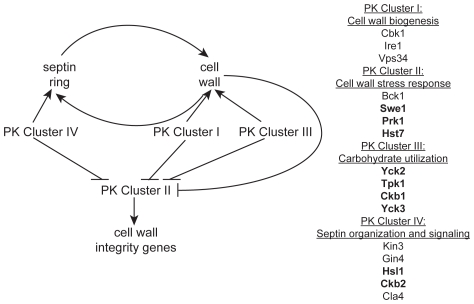
A model of the role of PKs in septin morphology and cell wall biogenesis. Based on our observations, an intact septin ring is required for normal cell wall production and a normal cell wall is required for the formation of a septin ring. PK Cluster IV genes have known and predicted roles in septin morphology and signaling and may impact cell wall biogenesis indirectly via this role. We hypothesize PK Cluster I genes have direct roles in biogenesis of the cell wall, while PK Cluster III may indirectly effect cell wall biogenesis by regulating the flow of carbohydrates into cell wall biosynthesis pathways. We hypothesize that the genes in PK Cluster II are involved in cell wall stress response and the upregulation of cell wall integrity genes. PK Clusters I, III, and IV negatively regulate PK Cluster II, either directly or indirectly, based on the observation that cell wall integrity genes are upregulated in the absence of these PKs.

PKs occupy key positions between environmental sensors and responses. Our study of *C. albicans* PKs emphasizes their pivotal roles: almost half of the PK mutants display a prominent phenotype. In contrast, only ∼5% of viable *C. albicans* transcription factor mutants have comparable phenotypes [16],[17],[31],[32]. We observed that *C. albicans* PK biological function is conserved with their *S. cerevisiae* and *S. pombe* orthologs in many cases (Tables 1 and 2). We also detected novel roles related to *C. albicans* filamentation and biofilm formation, traits not examined systematically with model organism mutant panels. However, our most striking result is that *C. albicans* CWR relies upon conserved PKs with novel functions compared to their orthologs, representing an expansion of the circuitry involved in this process (Fig. 5). A few of these PKs have been linked to cell wall integrity previously, most notably Prk1 and Hst7 [33]–[35], but their importance in CWR seems much greater in *C. albicans* than in *S. cerevisiae*, given that the orthologous *S. cerevisiae* mutants do not demonstrate heightened caspofungin sensitivity. CWR depends upon eight PKs with roles in this process that were not predicted from model yeast studies, plus a PK gene and a PK-related gene whose conserved CWR function is newly described in this report.

Two simple scenarios can account for the functional roles of the eight unpredicted CWR PKs: they may have a role in ongoing cell wall biogenesis, or they may only be required for response to cell wall stress. These possibilities are not mutually exclusive: a CWR PK may function in both capacities. Our analysis of PK mutant transcriptional responses strongly supports a role for Ire1 in normal cell wall biogenesis and suggests that several of the other novel PKs identified in this study may also have roles in cell wall biogenesis. In contrast, transcriptional responses of Cluster II PK mutants are not very distinct from wt and we suspect that these PKs may be important for cell wall stress response. Indeed, *BCK1*, a member of the cell wall stress response PKC1 MAPK pathway, and *PSK1*, which is also thought to have a role in cell wall stress response [17] are both in Cluster II. Microarray analysis of the other PKs in this cluster, Swe1 and Prk1, may identify a role for these PKs in stress response as well.

Our survey of gene expression alterations, though limited, revealed some qualitative differences among the mutants. Such distinctions may be useful in assembling the PKs into existing or novel pathways, a task that has generally proved challenging in organisms with limited genetic tractability. We were able to organize the data into distinct clusters that may reveal functional relationships. Indeed, mutants in *GIN4*, *CLA4*, and *HSL1*, genes important for the localization of septins (*GIN4* and *CLA4*) or signaling downstream of septin localization (*HSL1*), fell into a cluster (Cluster IV, Fig. 3). The *kin3−/−* and *ckb2−/−* mutants were also in this cluster. Although only tentatively linked, close association of the *kin3−/−* and *ckb2−/−* strains with mutants in genes involved in septin localization and signaling suggests Kin3 and Ckb2 may also play a role in this process (Fig. 5). Interestingly, the *cbk1−/−* and *ire1−/−* strains also clustered together (Cluster I, Fig. 2), lending further support to the hypothesis that *IRE1* has a role in cell wall biogenesis. Cbk1 is upstream of cell wall biogenesis genes, such as the chitin synthases [20], and along with the roles of Vps34 and Ire1 in cellular transport, this cluster seems to represent the production and delivery of cell wall building enzymes to the cell periphery (Fig. 5). Mutations in *SWE1*, *PRK1*, *PSK1*, and *HST7*, which fall into a cluster with the *bck1−/−* mutant (Cluster II, Fig. 2), clustered together because the genes tested in the QrtPCR analysis were not differentially regulated compared to wild type. Perhaps, like *bck1−/−*, they also have some role in responding to cell wall stress (Fig. 5). The third cluster includes both of the casein kinase I mutants (*yck2−/−* and *yck3−/−*), *tpk1−/−*, and the casein kinase II regulatory subunit mutant *ckb1−/−* (Fig. 2). The genes included in this cluster have a variety of roles within cells, but one intriguing correlation between their functions is carbohydrate sensing (Yck2/3) and signaling (Tpk1). The cAMP pathway, of which Tpk1 is a key member, has a conserved role in glucose sensing (reviewed in [36]) and Yck1 and 2 have been shown to have a role in regulation of glucose transporters [37]. Glucose is the major building block of the β-glucans in the cell wall as well as an important source of energy for cells and perhaps these genes help cells balance the need for food versus protection (Fig. 5). While such inferences are clearly tentative, they make numerous testable predictions that will be useful in unraveling the complex relationships among cell wall regulators.

Ire1 showed the largest damage profiles in all three cell wall damage response genes. In all organisms where it has been studied, Ire1 is a component of the conserved unfolded protein response (UPR), which is activated by accumulation of unfolded proteins in the ER [38]. *S. cerevisiae ire1* mutants do exhibit sensitivity to cell wall damage under certain conditions [39], but the *C. albicans ire1−/−* mutant seems much more sensitive to cell wall damage, suggesting perhaps a more central role in this process. A simple model is that the UPR is critical for *C. albicans* cell wall damage response, perhaps because of the large number of cell wall protein genes that are induced by caspofungin [16]. We note that a relatively distal 3′ homozygous insertion mutation was created for this study (Table S1; insertion at bp 3520 of the 3675 bp ORF), and we have been unable to generate homozygous 5′ insertions or a deletion of this gene in *C. albicans*. Thus the UPR may be essential in *C. albicans* even in the absence of exogenous stresses. We suggest that the UPR has a critical role in cell wall biogenesis and may be partially activated under our growth conditions.

Our findings also reveal a clear relationship between septin function and CWR. The PKs that govern CWR include Yck2, Cla4, and Gin4, which are involved in the assembly of septins at the mother-bud interface, and Swe1 and Hsl1, which are important for signaling that septins are appropriately localized [25],[40]. Furthermore, we observed that septins are required for normal sensitivity to caspofungin, and that septins are delocalized in response to caspofungin treatment. This phenomenon appears to be conserved in *S. cerevisiae* (Briggs, Mitchell, and Blankenship unpublished results), suggesting a conserved role for septin delocalization in cell wall integrity. Septins are connected to cell wall biogenesis through their known roles in determining sites of secretion and in directing the localization of chitin synthase. Septin delocalization may act positively in the cell wall damage response to permit delocalized secretion and cell wall synthesis when glucan synthase inhibition makes the bud neck an unsuitable site. A second possibility is that septin delocalization may function passively to prevent unbalanced production of cell wall components when glucan synthesis is blocked by caspofungin. A third possibility is that septin delocalization may protect cells by blocking cell cycle progression through Swe1 and Hsl1, thus reducing the need for new cell wall material. Thus, the analysis of *C. albicans* PKs has pointed toward a novel cell biological phenomenon, and several testable hypotheses may explain its functional consequences.

In some of our PK mutant strains, septins are mislocalized even in the absence of caspofungin treatment. This was expected for the *gin4−/−* mutant [29] and may contribute to the caspofungin sensitivity of this mutant strain, but we also identified several PKs with previously uncharacterized roles in septin organization. The *kin3−/−* strain clustered with mutants involved in septin organization and signaling (Class IV), and the septation defect of this mutant (Fig. 4) served as a validation of the clustering. Ckb2 was also a member of this cluster and although it did not have a septation defect, it may, like Hsl1, have a role in signaling downstream of septin organization. Septin mislocalization defects were also noted in the *cbk1−/−* and *vps34−/−* mutant strains. The *cbk1−/−* strain had septa at the mother-bud neck but also had aberrant septa in punctae throughout cells. Septa in *vps34−/−* mutant cells appeared normal in mature cells, but were wide in young buds, extending well into the bud, suggesting that septa in this mutant are disorganized at early stages of bud formation. Both of these mutants are in Cluster I, and perhaps the mislocalization of septins in these strains is due to the damaged walls present in these mutants. The *cbk1−/−* and *vps34−/−* phenotypes are different and the *ire1−/−* mutant does not have a septin localization defect at all, suggesting that the nature of the cell wall defect in these strains are distinct. We propose that Class I PKs are important for cell wall biogenesis and that defects in biogenesis or defects due to external stress inactivate Class IV PKs (perhaps via Class II PKs), which leads to septin mislocalization.

Several studies have pointed toward extensive rewiring of *C. albicans* transcriptional regulatory pathways. There are two main kinds of phenomena. First, for many orthologous *C. albicans*-*S. cerevisiae* transcription factors, both the nature of target genes and the signals to which they respond are distinct in each organism [41]–[44]. Second, *C. albicans* has some functionally significant transcriptional regulators, such as Mtl**a**2 or Cas5, that do not have clear *S. cerevisiae* orthologs [16],[45]. In contrast, many of the relationships among *C. albicans* PKs and the cell wall seem to reflect not “rewiring” but perhaps a greater amount of “current” in *C. albicans*. That is, one can develop plausible mechanistic explanations for the *C. albicans* PKs implicated in CWR, based upon the known functions of their orthologs in *S. cerevisiae* and the assumption that there is greater flux through, or stress upon, the *C. albicans* secretory system. Ire1 and the septin regulatory network are cases in point. However, we also find indications of true PK rewiring. The MAPKKK Hst7, which functions in the *C. albicans* mating response-filamentous growth pathway similarly to its *S. cerevisiae* ortholog Ste7 [46]–[49], seems to have acquired an adjunct function in cell wall biosynthesis or integrity. Similarly, the connection of CWR to Kis1 and Sip3, two proteins whose *S. cerevisiae* orthologs interact with the glucose repression regulatory PK Snf1 [50],[51], suggests that the *C. albicans* glucose repression system may be connected to cell wall biogenesis or integrity. Although a *S. cerevisiae snf1* mutant shows synthetic lethality with the β-1,3-glucan synthase *FKS1* [52] we propose that this PK has a more prominent role in CWR in *C. albicans* and may explain why it is essential for viability in this fungus [53],[54]. Caspofungin hypersensitivity of mutants defective in Rio2, Tpk1, and casein kinase 2 regulatory subunits Ckb1 and Ckb2 also suggests that PK rewiring has occurred during divergence of *S. cerevisiae* and *C. albicans*. These cases provide a foundation for further mechanistic inquiry into cell wall regulatory responses of this highly successful commensal and pathogen.

## Materials and Methods

### Strains and media

Strains were grown on yeast extract-peptone-dextrose (YPD) rich medium, defined synthetic dextrose medium, prepared as previously described [55], spider medium (10 g D-mannitol (Sigma), 10 g nutrient broth (BD Difco), 2 g K_2_HPO_4_ (Sigma) in 1 l of H_2_O), and M199 cell culture medium (Roche) buffered with 150 mM Hepes (Gibco). NaCl (Sigma), H_2_O_2_ (Sigma), Caspofungin (Merck), and sorbitol (Sigma) were added to media at the concentrations described. M199 medium was adjusted to the appropriate pH using NaOH and HCl, and pH was measured on a Corning pH Meter 240.

All PK mutants tested were made in the BWP17 [56] background. Construction of the PK mutant library followed the methods established in Davis et al [57]. Briefly, clones bearing genomic DNA from *C. albicans* strain CAI4 with an identified insertion of Tn7-*UAU1* cassette were excised from the plasmid backbone by digestion with NotI. These linearized constructs were then transformed into BWP17. Putative heterozygote (Arg+) transformants were selected on SC-Arg+Uridine plates, and twelve independent colonies were grown in YPD liquid media overnight and patched onto SC-Arg-Ura plates. The Arg+ Ura+ putative homozygotes were screened by colony PCR using primers ∼500 bp up and downstream of the insertion and within the transformant cassette (Arg4detect [32]) to ensure absence of the wild type allele and presence of the transformed alleles. A BWP17 colony was amplified with the same primer set as a control. These strains have been deposited and are available at the Fungal Genetics Stock Center http://www.fgsc.net/candida/FGSCcandidaresources.htm (Kansas City, MO).

To complement specific deletions, a fragment of DNA∼800 bp upstream and 200 bp downstream of the open reading frame was amplified from BWP17 genomic DNA. Primers for the complementation were roughly 60–70 bp in length and the 3′ 20–30 bp was gene specific. A 40mer sequence (upstream primer-TTCACACAGGAAACAGCTATGACCATGATTACGCCAAGCT, downstream primer TCGACCATATGGGAGAGCTCCCAACGCGTTGGATGCATAG) was tacked onto the 5′ end of each primer sequence to guide *in vivo* recombination into the pDDB78 plasmid [58]. The amplified complementation fragment was co-transformed into the *S. cerevisiae* BY4741 Δ*trp*1 strain with an EcoRI/NotI linearized pDDB78 plasmid, and the resulting complementation clone was amplified in *E. coli*. Each complement clone was digested with NruI to target insertion to the *HIS1* locus and transformed into the appropriate mutant strains, complementing the mutation and rendering the strains His+ as well. Presence of a wild-type band was assayed by colony PCR using the complement primers. pDDB78, digested with NruI, was transformed into the same mutant strains to generate prototrophic, marker-matched strains for comparison with the complemented strains.

To insert GFP into the *SEP7* locus in *his1−/−* PK mutant and wild type (DAY286) *his1−/−* strains, a fragment of the *SEP7* gene was amplified from the YSM26-1 strain [28] containing the 3′ end of the *SEP7* gene and the N-terminal GFP tag. The same strategy used for creating the complementation clones was utilized to make a *HIS1*-tagged *SEP7-GFP* integrating plasmid, generating pJRB103. This plasmid was digested with BclI (New England Biolabs), which cuts within the *SEP7* sequence to direct integration at the native *SEP7* locus. Integration of the construct into the native *SEP7* locus was assayed by PCR using primers within the *GFP* sequence and upstream of the *SEP7* integration.

### Identification of PK genes

A total of 108 PK were identified by scanning *C. albicans* genome databases (CGD [59] and CandidaDB [60]) for genes annotated as PKs as well as searching for *S. cerevisiae* and *S. pombe* PK homologs by blastp analysis (Table S1). Identities between *C. albicans* PKs and putative *S. cerevisiae* orthologs ranged from 23%–83% and putative *S. pombe* orthologs from 22%–78% (although in four instances, the best matches with *S. pombe* were not PKs). An additional 13 genes, identified as PK-related, were also included in subsequent analyses.

### Sensitivity assays

Osmotic, oxidative, pH, caspofungin sensitivity. Strains were grown overnight in YPD media at 30°C. Cell density was measured at OD_600_ for each strain, which were then diluted to an OD_600_ of 3 in H_2_O. Five-fold dilutions were made of the OD_600_ 3 stock and these were plated on the indicated media. Plates were incubated at 30°C for 24–48 hours, and digitally photographed. When sensitivity was identified in *C. albicans*, orthologous a and α *S. cerevisiae* mutants from the *S. cerevisiae* mutant library [61] were also assayed for sensitivity in a similar manner.

### Filamentation and biofilm assays

For filamentation assays, overnight cultures of strains were diluted to an OD_600_ of 0.3 in M199 liquid medium. Cultures were incubated at 37°C with shaking for 90 minutes and cells were examined under the microscope (see below).

Biofilm assays were performed as previously described [31]. Briefly, silicon disks were incubated at 37°C overnight in a 12-well plate with bovine serum (Sigma) and washed with 2 ml PBS. 2 ml of Spider medium was added to each well. Aliquots of overnight cultures were added to an OD_600_ of 0.5 to each well, and incubated with the pre-treated silicon disks for 90 minutes at 37°C with light shaking. Following this adherence step, the silicon disks were carefully transferred to a new 12-well plate with 2 ml of PBS in each well and then to another 12-well plate with fresh spider medium. Cultures were incubated at 37°C with light shaking for 60 hours and then digitally imaged.

### RNA collection and quantitative rtPCR

Overnight cultures of cells were diluted to an OD_600_ of 0.2 in 50 ml fresh YPD medium. Cultures were allowed to grow at 30°C with shaking until culture density reached an OD_600_ of ∼1. Cells for the untreated assay were then harvested by vacuum filtration and flash frozen in a dry ice/EtOH bath. Cultures for the 30 minute caspofungin treatment assay were split into two cultures after reaching an OD_600_ of ∼1. 125 ng/ml caspofungin was added to one culture, an equal volume of water was added to the other, and the cultures were allowed to incubate for 30 minutes before harvesting. Strains carrying homozygous mutants of *YCK2*, *YCK3*, *RIO2*, and *PRK1* were generated after the completion of the treatment experiment and were thus not included in the final assay. Cells were kept frozen on filters at −80°C until RNA extraction. RNA was extracted using a RiboPure-Yeast kit (Ambion) following manufacturers instructions with the following modifications. Cells were resuspended from filters with 1.5 ml ice-cold dH_2_O followed by 10–15 seconds of vigorous vortexing. Resuspended cells were transferred to a 1.5 ml tube and spun down following the manufacturers protocol. Furthermore, during the cell disruption step, cells were beaten with a Next Advance Bullet Blender for 3 min at 4°C to maximize cell lysis.

10 µg of the resulting total RNA was treated with the DNA-free kit (Ambion) followed by first-strand cDNA synthesis from half of the DNA-free RNA using the AffinityScript multiple temperature cDNA synthesis kit (Stratagene). Absence of DNA contamination was confirmed using control sets for which reverse transcriptase was omitted from the cDNA reaction.

Primer3 software (http://frodo.wi.mit.edu/) was used to design primers for four genes, *TDH3*, *DDR48*, *SOD5*, and *STP4*. The primers were as follows: for *TDH3*, TDH3FOR, 5′-ATCCCACAAGGACTGGAGA-3′, and TDH3REV, 5′-GCAGAAGCTTTAGCAACGTG-3′; for *DDR48*, JRB212, 5′-TTTCGGTTTCGGTAAAGACG-3′, and JRB213, 5′-CTGTTGGAGGAACCGTAGGA-3′; for *SOD5*, JRB249, 5′-TCCTGCTGCTCATGAAGTTG-3′, and JRB250, 5′-TTGTGTTAGCATTGCCGTGT-3′; and for *STP4*, JRB244, 5′-TCCTTTCAAGAACATCGATTCA-3′, and JRB245, 5′-TTATGCATCCAATCATCGACA-3′. 2× iQ SYBR Green Supermix (Biorad), 1 µl of first-strand cDNA reaction mixture, and 0.1 µM of primers were mixed in a total volume of 50 µl per reaction, and real-time PCR was performed in triplicate for each sample on an iCycler iQ real-time PCR detection system (Bio-Rad). The program for amplification had an initial denaturation step at 95°C for 5 min, followed by 40 cycles of 95°C for 45 s and 58°C for 30 s. Product amplification was detected using SYBR Green fluorescence during the 58°C step, and specificity of the reaction was monitored by melt-curve analysis following the real-time program. *TDH3* was used as a reference gene for normalization of gene expression, which was determined using Bio-Rad iQ5 software (ΔΔ*C_T_* method). Cluster analysis was performed with the Multiexperiment Viewer (MeV 4.3) from TIGR. Hierarchical clustering was performed with average linkage and a Manhattan distance metric.

### Septin morphology and microscopy

*SEP7-GFP* cells were diluted and grown overnight to an OD_600_ of ∼1 in YPD. 200 µl of this culture was pipeted onto glass bottom dishes (MatTek Corporation, Ashalnd, MA) coated with concanavalin A or poly-D-lysine (for caspofungin treatment). The dishes were washed twice with PBS and resuspended with PBS for immediate visualization (untreated cells) or incubated with 125 ng/ml caspofungin in YPD for 30 minutes (treated cells). Following caspofungin treatment, the dishes were washed again and PBS was added for visualization. Cells visualized with a Zeiss Axio Observer Z.1 fluorescence microscope and a 100× NA 1.4 objective. Fluorescent images were acquired with an exposure time of 1 s on a Coolsnap HQ^2^ (Photometrics) camera using Axiovision (Zeiss) software.

## Supporting Information

Figure S1 PK and PK-related genes play essential roles in survival of and response to common *in vivo* stresses. (A) A wild type marker-matched strain (DAY286) and strains mutant for *hog1/hog1* (JMR115), *pbs2/pbs2* (JJH31), *chk1/chk1* (JJH33), *sln1/sln1* (SF040), *prk1/prk1* (JJH50), *kin4/kin4* (SF021), *ssn3/ssn3* (JJH65), *ste11/ste11* (SF041A), *gin4/gin4* (JJH87), *ckb1/ckb1* (SF039), *kis1/kis1* (JJH85), and *ssn8/ssn98* (SF046) were serially diluted on YPD (−) or YPD + 7mM H_2_O_2_ and grown for 2 days at 30°C. (B) DAY286 (WT) and strains mutant for *hog1/hog1, pbs2/pbs2, sok1/sok1* (JJH106), *gin4/gin4*, and *kis1/kis1* were serially diluted on YPD (−) or YPD + 1.5M H_2_O_2_ and grown for 2 days at 30°C. (C) DAY286 (WT) and a strain mutant for *cbk1/cbk1* (JJH114) were serially diluted on M199 solid medium buffered at pH 7 or pH 4 and grown for 2 days at 30°C. For all assays, results shown are representative of 2 or more independently isolated strains where possible. (D) A wild type strain (DAY286) and strains mutant for *ire1/ire1* (SF008A), *sok1/sok1* (JJH104), *mss2/mss2* (JJH93), *tpk2/tpk2* (SF026), *cbk1/cbk1*, and *rio2/rio2* (JJH243) were grown overnight in rich liquid medium (YPD), diluted, and then grown in either liquid YPD at 30°C with shaking, or in M199 cell culture medium at 37°C for 2 hours and then imaged. (E) DAY286 (WT), a *bcr1Δ/Δ* negative control (CJN702), and strains mutant for *cbk1/cbk1, ire1/ire1*, and *gin4/gin4* (JJH87) were tested for biofilm formation in spider medium. Biofilms were allowed to mature for 48 hours before imaging. In both assays, results are representative of 2 isolates where possible. Only those PK and PK-related mutant strains with distinct phenotypes from wt are shown in this figure.
(1.52 MB PDF)

Figure S2 Expression profiles of PK mutants in the absence of stress. The expression of *ALS1, STP4, SOD5, DDR48, RTA4*, and *ECM331* were measured in caspofungin-sensitive PK and PK-related mutant strains and their respective complement strains. All strains were grown in rich media at 30°C in the absence of exogenous stressors. The expression of *TDH3* was used to normalize expression between strains and all expression was compared to a marker-matched wild type strain (DAY185). The panels are divided into the clusters identified in Fig. 2. (A) Cluster I, (B) Cluster II, (C) Cluster III, and (C) Cluster IV. Error bars represent standard deviation from the mean.
(0.32 MB PDF)

Figure S3 Septins are mislocalized in a subset of PK mutants. The localization of SEP7-GFP was monitored in PK mutants sensitive to cell wall stress. Strains were grown in YPD without additional stress. Images shown are representative images from a panel of images taken of each strain. Strains are as follows: *bck1−/−* (JRB194), *cbk1−/−* (JRB224), *ckb1−/−* (JRB198), *ckb2−/−* (JRB229), *cla4−/−* (JRB183), *gin4−/−* (JRB221), *hsl1−/−* (JRB188), *ire1−/−* (JRB227), *kin3−/−* (JRB193), *kis1−/−* (JRB167), *mkc1−/−* (JRB208), *mkk2−/−* (JRB205), *mss2−/−* (JRB177), *pkc1−/−* (JRB200), *prk1−/−* (JRB169), *rio2−/−* (JRB225), *sip3−/−* (JRB170), *swe1−/−* (JRB179), *tpk1−/−* (JRB174), *vps34−/−* (JRB216), *yck2−/−* (JRB164), *yck3−/−* (JRB212).
(4.38 MB PDF)

Table S1 Genetic and biological properties of *C. albicans* insertion mutants.
(0.07 MB XLS)
