# Supplementary figures and images for: An Extensive Circuitry for Cell Wall Regulation in Candida albicans
Source: PLoS Pathog. 2010 Feb 5;6(2):e1000752. doi: 10.1371/journal.ppat.1000752 (PMC2816693; doi:10.1371/journal.ppat.1000752)

Fig S1

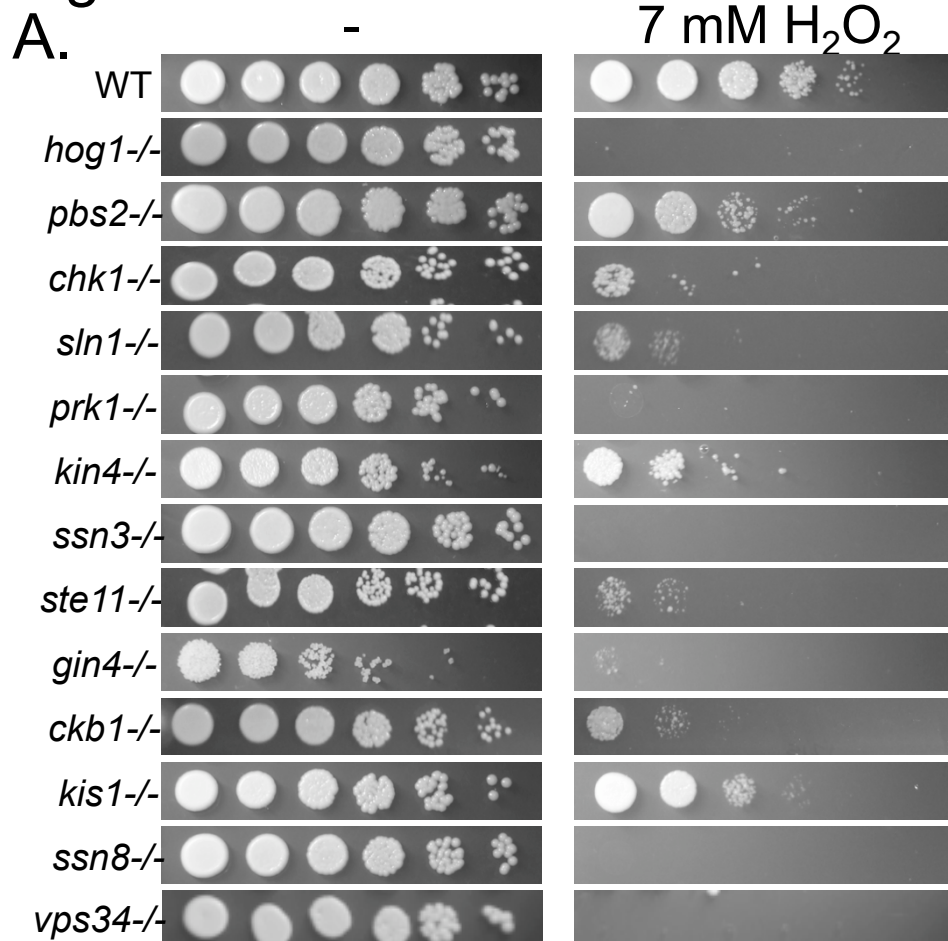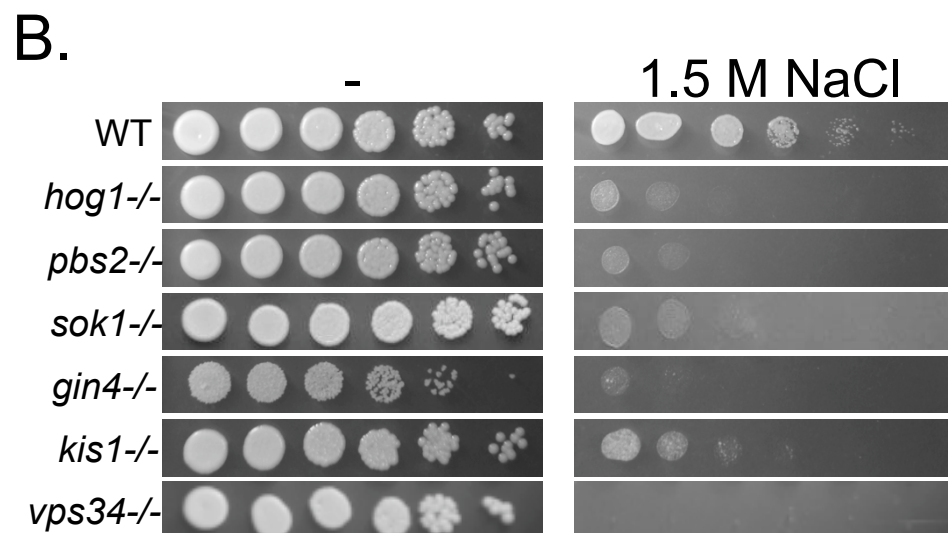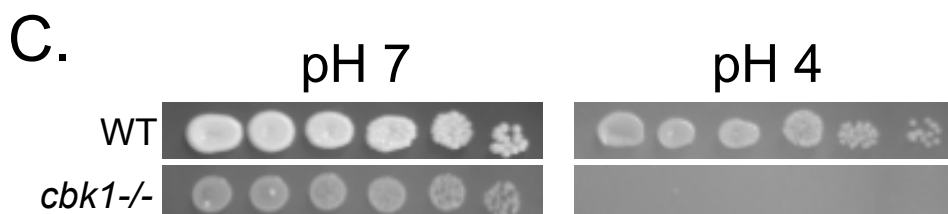

Figure S1 cont...

D.

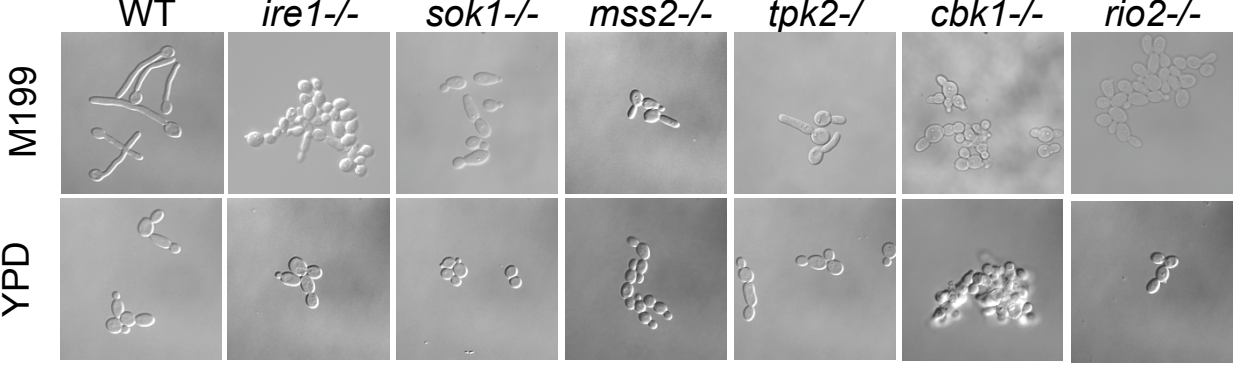

E.

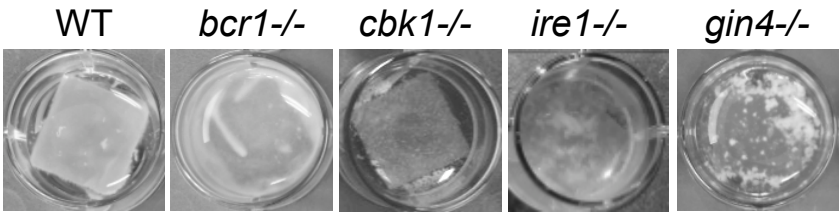

Supplement: Figure S1 — PK and PK-related genes play essential roles in survival of and response to common in vivo stresses. (A) A wild type marker-matched strain (DAY286) and strains mutant for hog1/hog1 (JMR115), pbs2/pbs2 (JJH31), chk1/chk1 (JJH33), sln1/sln1 (SF040), prk1/prk1 (JJH50), kin4/kin4 (SF021), ssn3/ssn3 (JJH65), ste11/ste11 (SF041A), gin4/gin4 (JJH87), ckb1/ckb1 (SF039), kis1/kis1 (JJH85), and ssn8/ssn98 (SF046) were serially diluted on YPD (−) or YPD + 7mM H2O2 and grown for 2 days at 30°C. (B) DAY286 (WT) and strains mutant for hog1/hog1, pbs2/pbs2, sok1/sok1 (JJH106), gin4/gin4, and kis1/kis1 were serially diluted on YPD (−) or YPD + 1.5M H2O2 and grown for 2 days at 30°C. (C) DAY286 (WT) and a strain mutant for cbk1/cbk1 (JJH114) were serially diluted on M199 solid medium buffered at pH 7 or pH 4 and grown for 2 days at 30°C. For all assays, results shown are representative of 2 or more independently isolated strains where possible. (D) A wild type strain (DAY286) and strains mutant for ire1/ire1 (SF008A), sok1/sok1 (JJH104), mss2/mss2 (JJH93), tpk2/tpk2 (SF026), cbk1/cbk1, and rio2/rio2 (JJH243) were grown overnight in rich liquid medium (YPD), diluted, and then grown in either liquid YPD at 30°C with shaking, or in M199 cell culture medium at 37°C for 2 hours and then imaged. (E) DAY286 (WT), a bcr1Δ/Δ negative control (CJN702), and strains mutant for cbk1/cbk1, ire1/ire1, and gin4/gin4 (JJH87) were tested for biofilm formation in spider medium. Biofilms were allowed to mature for 48 hours before imaging. In both assays, results are representative of 2 isolates where possible. Only those PK and PK-related mutant strains with distinct phenotypes from wt are shown in this figure. (1.52 MB PDF) [file ppat.1000752.s001.pdf]

Figure S2

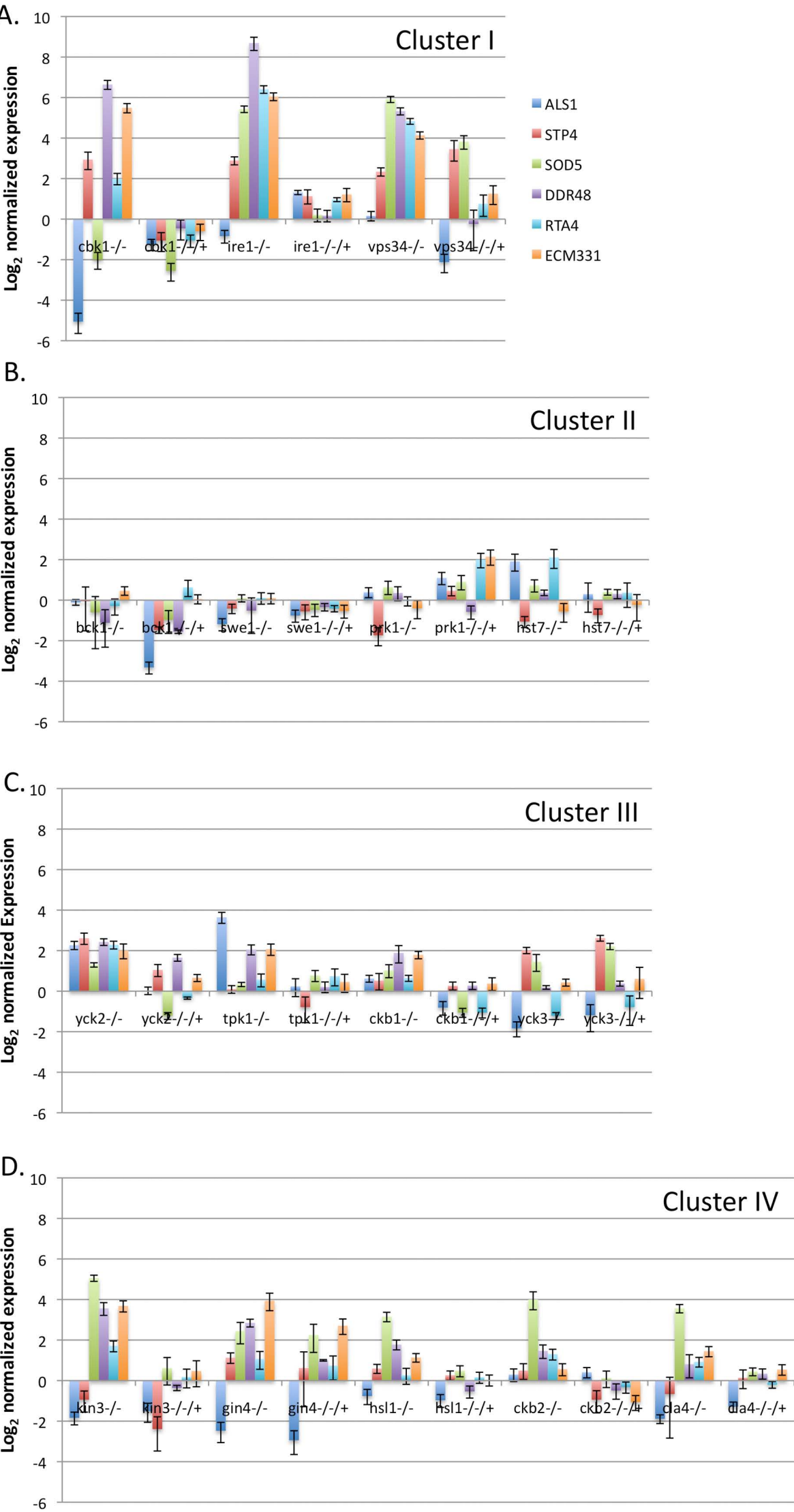

Supplement: Figure S2 — Expression profiles of PK mutants in the absence of stress. The expression of ALS1, STP4, SOD5, DDR48, RTA4, and ECM331 were measured in caspofungin-sensitive PK and PK-related mutant strains and their respective complement strains. All strains were grown in rich media at 30°C in the absence of exogenous stressors. The expression of TDH3 was used to normalize expression between strains and all expression was compared to a marker-matched wild type strain (DAY185). The panels are divided into the clusters identified in Fig. 2. (A) Cluster I, (B) Cluster II, (C) Cluster III, and (C) Cluster IV. Error bars represent standard deviation from the mean. (0.32 MB PDF) [file ppat.1000752.s002.pdf]
