# Supplementary material for: An Extensive Circuitry for Cell Wall Regulation in Candida albicans
Source: PLoS Pathog. 2010 Feb 5;6(2):e1000752. doi: 10.1371/journal.ppat.1000752 (PMC2816693; doi:10.1371/journal.ppat.1000752)
Supplement: Figure S3 — Septins are mislocalized in a subset of PK mutants. The localization of SEP7-GFP was monitored in PK mutants sensitive to cell wall stress. Strains were grown in YPD without additional stress. Images shown are representative images from a panel of images taken of each strain. Strains are as follows: bck1−/− (JRB194), cbk1−/− (JRB224), ckb1−/− (JRB198), ckb2−/− (JRB229), cla4−/− (JRB183), gin4−/− (JRB221), hsl1−/− (JRB188), ire1−/− (JRB227), kin3−/− (JRB193), kis1−/− (JRB167), mkc1−/− (JRB208), mkk2−/− (JRB205), mss2−/− (JRB177), pkc1−/− (JRB200), prk1−/− (JRB169), rio2−/− (JRB225), sip3−/− (JRB170), swe1−/− (JRB179), tpk1−/− (JRB174), vps34−/− (JRB216), yck2−/− (JRB164), yck3−/− (JRB212). (4.38 MB PDF) [file ppat.1000752.s003.pdf]

Figure S3

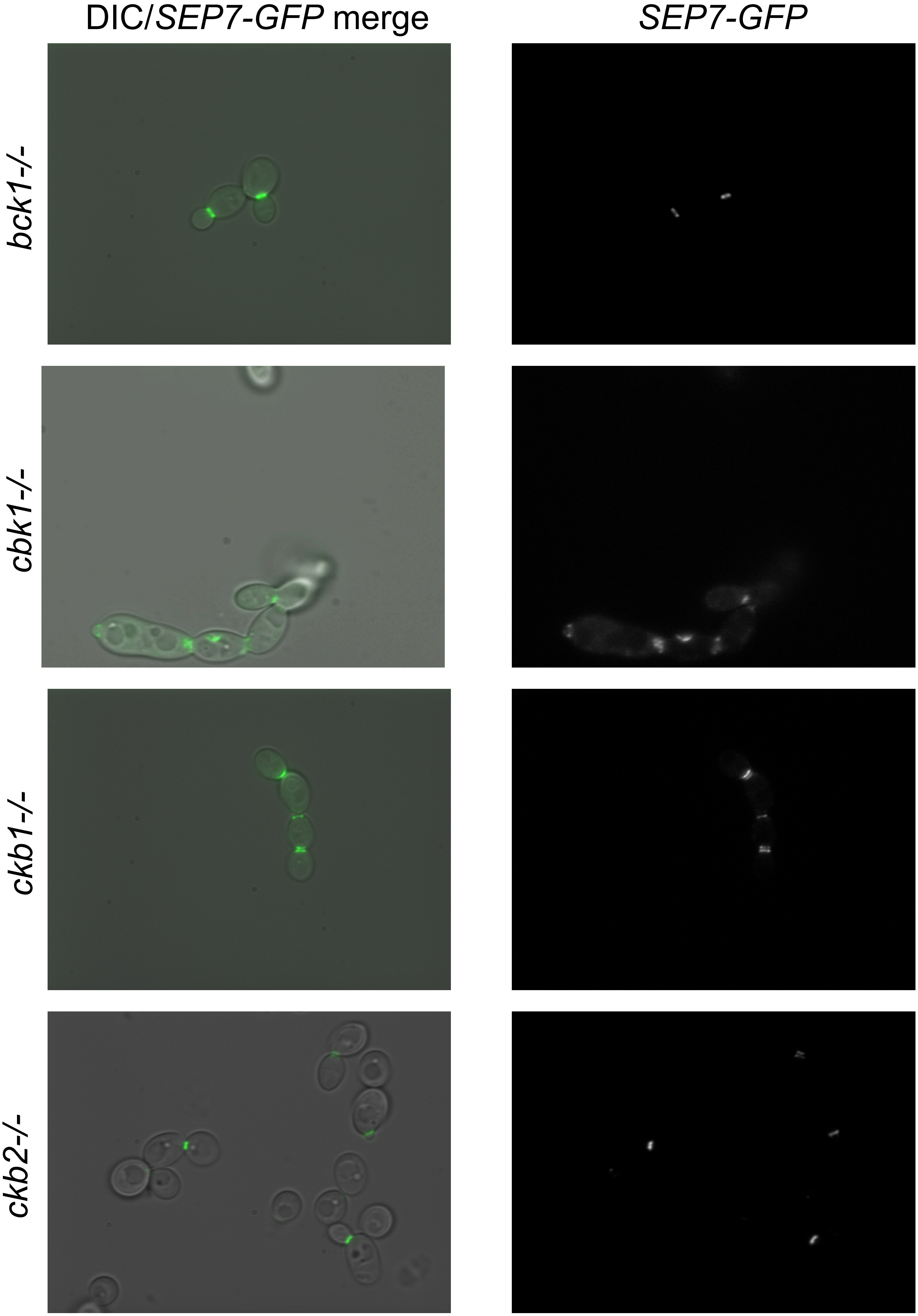

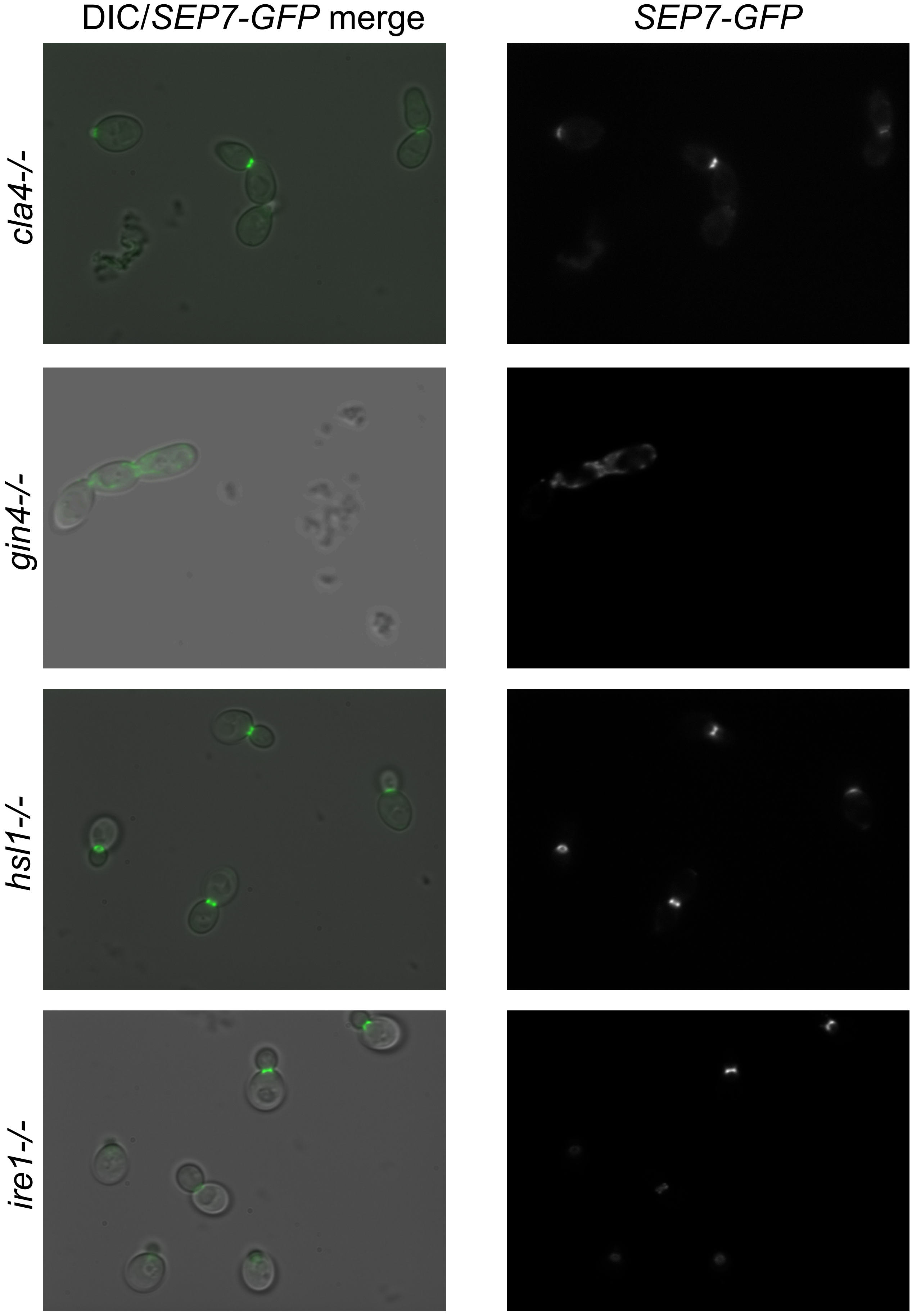
DIC/SEP7-GFP merge
SEP7-GFP
cla4-/-
gin4-/-
hsl1-/-
ire1-/-

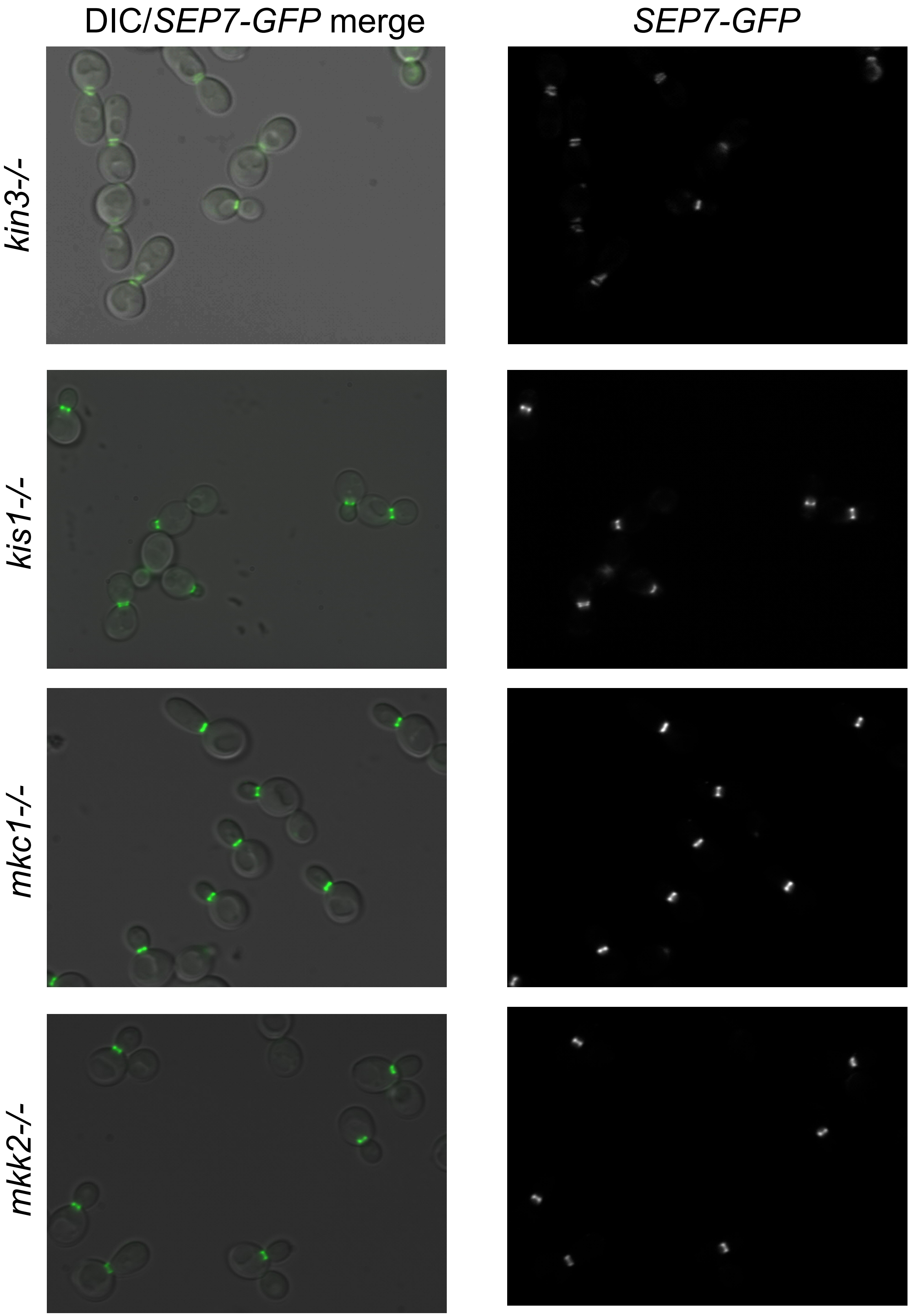
DIC/SEP7-GFP merge
SEP7-GFP
kin3-/-
kis1-/-
mkc1-/-
mkk2-/-

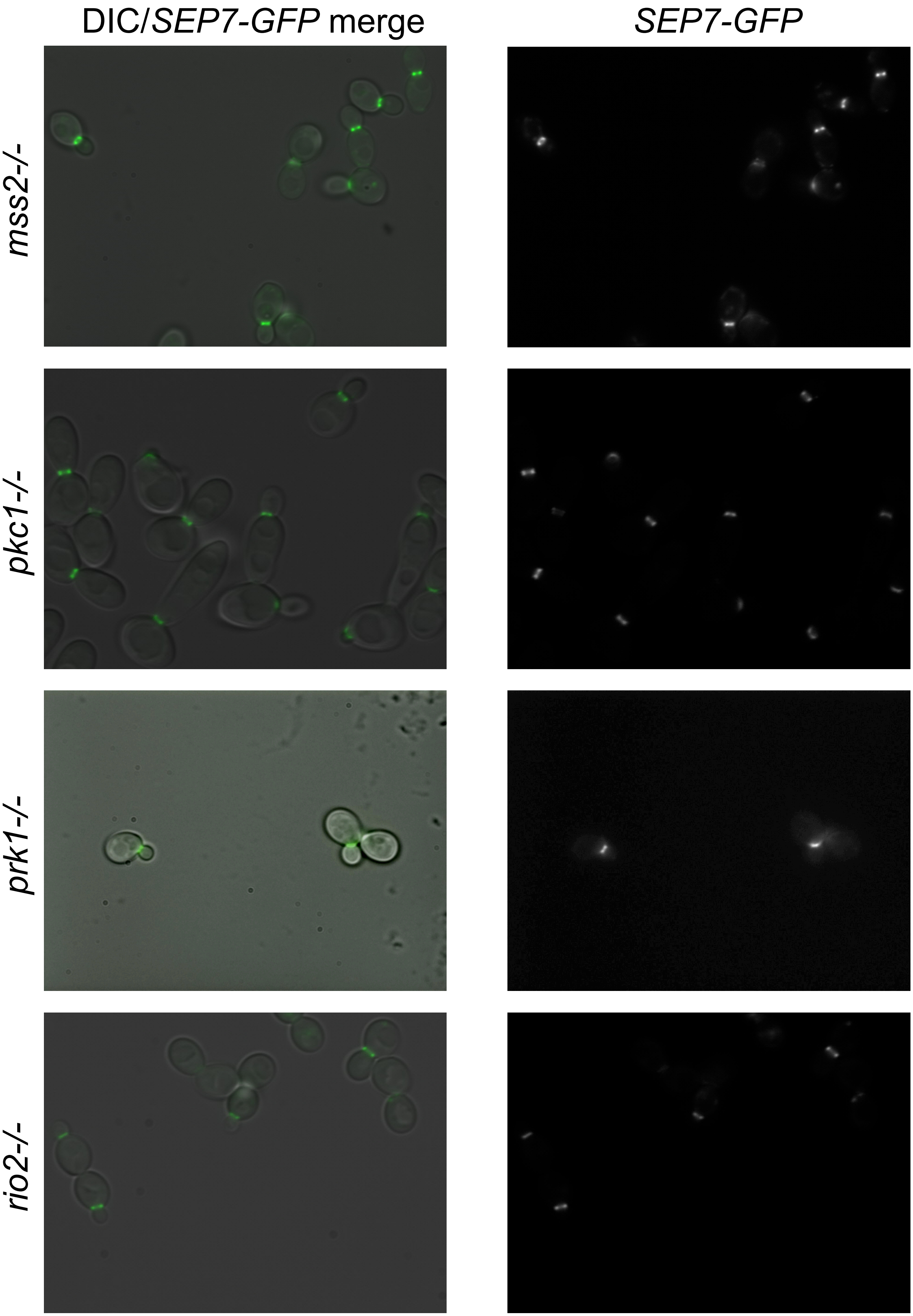

DIC/*SEP7-GFP* merge
SEP7-GFP
mss2-/-
pkc1-/-
prk1-/-
rio2-/-

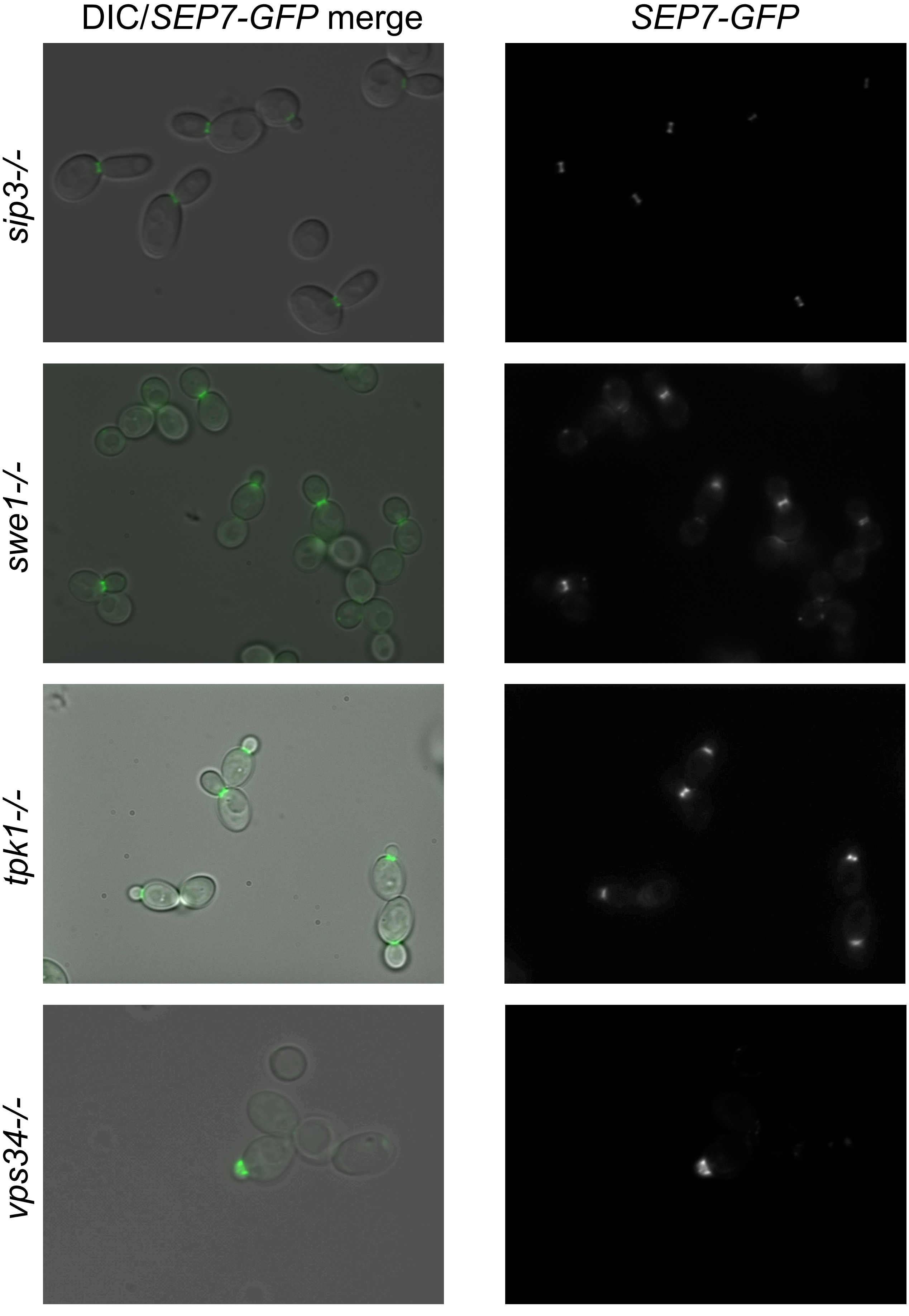

DIC/*SEP7-GFP* merge
SEP7-GFP
sip3-/-
swe1-/-
tpk1-/-
vps34-/-

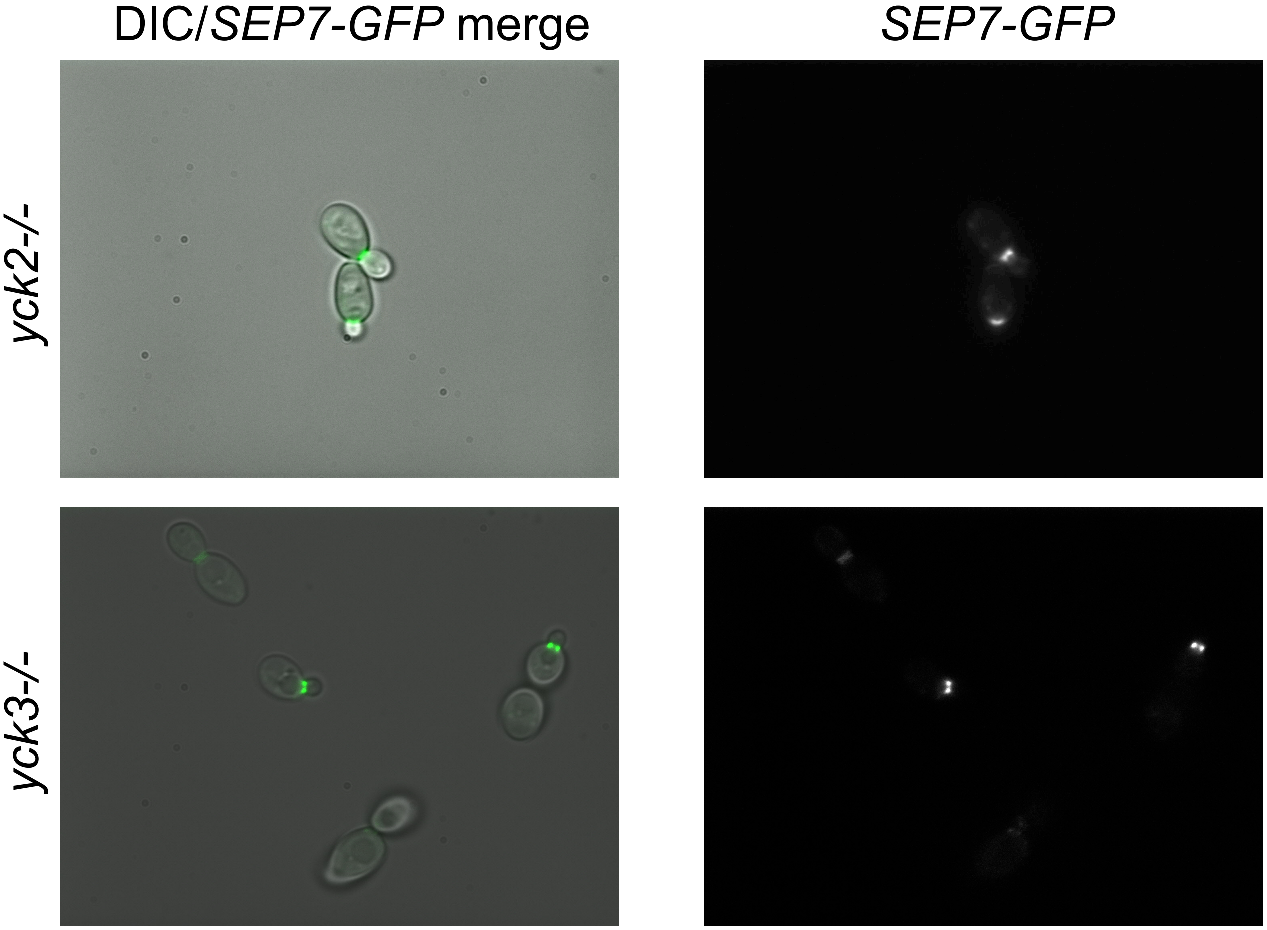

DIC/*SEP7-GFP* merge
SEP7-GFP
yck2-/-
yck3-/-
